# Communal nursing in wild house mice is not a by-product of group living: Females choose

**DOI:** 10.1007/s00114-013-1130-6

**Published:** 2014-01-04

**Authors:** Andrea Weidt, Anna K. Lindholm, Barbara König

**Affiliations:** Institute of Evolutionary Biology and Environmental Studies, University of Zurich, Winterthurerstrasse 190, 8057 Zurich, Switzerland

**Keywords:** Cooperation, Plural breeding, House mouse, Communal nesting, Partner choice

## Abstract

Communal nursing, the provision of milk to non-offspring, has been argued to be a non-adaptive by-product of group living. We used 2 years of field data from a wild house mouse population to investigate this question. Communal nursing never occurred among females that previously lacked overlap in nest box use. Females nursed communally in only 33 % of cases in which there was a communal nursing partner available from the same social group. Solitarily nursing females were not socially isolated in their group; nevertheless, high spatial associations prior to reproduction predict which potential female partner was chosen for communal nursing. An increase in partner availability increased the probability of communal nursing, but population density itself had a negative effect, which may reflect increased female reproductive competition during summer. These results argue that females are selective in their choice of nursing partners and provide further support that communal nursing with the right partner is adaptive.

**Electronic supplementary material:**

The online version of this article (doi:10.1007/s00114-013-1130-6) contains supplementary material, which is available to authorized users.

## Introduction

A controversial example of altruism in mammals is communal nursing, where milk is shared between own pups and young produced by another mother. Communal nursing occurs in all major mammalian taxa and is relatively common among rodents, carnivores and pigs (Packer et al. 1992). Some authors consider it a non-adaptive trait as lactation in mammals involves high energetic investment and can result in future reproductive costs (see Lewis and Pusey 1997; Hayes 2000; Roulin 2002). The nursing of non-offspring thus benefits others to the detriment of the lactating female. In lions, non-offspring nursing have been interpreted as an unavoidable by-product of group living (Pusey and Packer 1994). Manning et al. (1992) observed, in their study of house mice in semi-natural enclosures, that solitary nests mainly occurred when there was no opportunity to nest communally. Therefore, they suggested that communal nursing is a side effect of sharing the same nest. Similarly, in deer mice, communal nursing has been associated with high-population density and lack of opportunity for dispersal (Wolff 1994). Other non-adaptive explanations of non-offspring nursing are milk theft by parasitic young, misdirected maternal care or sexual conflict (for reviews see Lewis and Pusey 1997; Hayes 2000; Roulin 2002; Roulin and Hager 2003).

The alternative view is that communal nursing is an adaptive trait. Laboratory studies on mice and rats describe improved growth or survival of own offspring (Sayler and Salmon 1969; Mennella et al. 1990; Heiderstadt and Blizard 2011), and even improved lifetime reproductive success for females that nurse communally (König 1993, 1994a).

In house mice (*Mus musculus domesticus*), females of the same social group may pool litters in a communal nest where they nurse their own and non-offspring. This has been documented for inbred and wild females kept in the laboratory, in large enclosures and under natural conditions in the field (e.g. Southwick 1955; Sayler and Salmon 1969; Wilkinson and Baker 1988; König 1993; Manning et al. 1995). Laboratory studies with wild house mice show mutualistic and direct fitness benefits of communal nursing, which are modified by familiarity and by group size (König 1994a, b). Indeed, females display non-random preferences for social partners when kept in enclosures. Such spatial associations between females in nest boxes were used to select pairs of females with positive or with no preferences. Those pairs with positive preferences, when housed together later, had a significantly higher lifetime reproductive success than pairs that had no preference for each other (Weidt et al. 2008).

Here, in a study of wild mice from the same strain used in Weidt et al. (2008), we analyse the pattern of communal vs. solitary nursing in individually marked females in a free-ranging population over 2 years. Both adaptive and non-adaptive hypotheses predict that females choose a communal nursing partner only from those using common sleeping and breeding nests and thus belonging to the same social group. The non-adaptive hypothesis predicts that females will communally nurse with any female with a litter from the same social group. It also predicts that the incidence of communal nursing will increase with population density. By contrast, the adaptive hypothesis predicts that females exhibit social partner choice, and that such choice can be foreseen by spatial associations of females before giving birth.

## Materials and methods

In 2002, we established a population of free-ranging wild house mice in a 72-m^2^ barn outside Zurich, Switzerland, equipped with 40 nest boxes (König and Lindholm 2012). We monitored the population continuously from November 2002 until December 2004. At weekly intervals, we weighed each mouse, visually assessed reproductive status, and recorded any pups present in nest boxes. Mice weighing at least 18 g were tagged with Trovan® transponders for individual identification. The nesting pattern of all tagged individuals was determined 3–5 times per week by scanning nest boxes using a portable transponder-reader. To assign motherhood, we considered signs of lactation vs. pregnancy, a reduction in body weight after birth, and proximity to pups.

### Communal nests and communal nursing options

Once pups of different litters are placed in one nest, females nurse them indiscriminately, and are unable to tell own pups apart from non-offspring (König 1989; König 2006; but see Hager and Johnstone 2005 for an inbred strain). It can thus be safely assumed that communal nursing takes place whenever different litters are combined in one nest. At day 17, pups are mobile and begin to consume solid food. We therefore defined communal nests as nests containing two or more litters differing in development, below the age of 17 days.

Potential communal nursing partners (referred to as communal nursing options) for a female required the following: (1) temporal synchrony, defined as females that reproduced up to 16 days prior to the birth of the focal female's litter and (2) spatial synchrony, defined as overlap in use of at least one common nest box (shared home range; also see Supplementary Material). We asked whether spatial synchrony in nest box use during the non-reproductive period predicted communal nursing. We calculated dyadic associations according to the symmetrical index of Fager (Kerth and König 1999; see Supplementary Material) during the females' non-reproductive period. We compared dyadic associations of two females that communally nursed with dyadic associations of a mother that nursed alone and her potential, but not chosen nursing partner. If a female had more than one communal nursing option, we took the mean of the indices. Also, if a female contributed her litter to a communal nest already consisting of two or more litters, we averaged the Fager's indices of the communal nursing partners.

Finally, we carried out a generalized linear model to test the effect of number of communal nursing options, population density (number of adult mice per square metre, based on population monitoring for the month when the litter was born), primiparity (first birth), post-partum gestation (lactation of the previous litter while gestating the current litter) and season of birth date of the litter (summer vs. winter, with 1 March to 31 August defined as summer) on the propensity to communally nurse. Statistics were conducted using R Version 2.12.2 (R Development Core Team 2011).

## Results

### Communal nursing options and decisions

Over 2 years, 106 litters with 526 pups were born in our study population (see Supplementary Material). In 61 of 106 litters, the mother had the option to nurse communally, meaning that another litter of maximally 16 days of age was available in the mother's home range. Individual mothers had between one and five options to establish a communal nest. A female used one of these options in 20 cases (33 %). For further analyses, we only used the 49 cases (*N* = 31 females) where maternity could be fully resolved, leaving 16 cases where females chose to rear a litter communally (*N* = 16 females), and 33 (*N* = 23 females) where females did not use any of the available options. Eight of the 31 females reared offspring both solitarily and communally.

### Individual associations

**Fig. 1:**
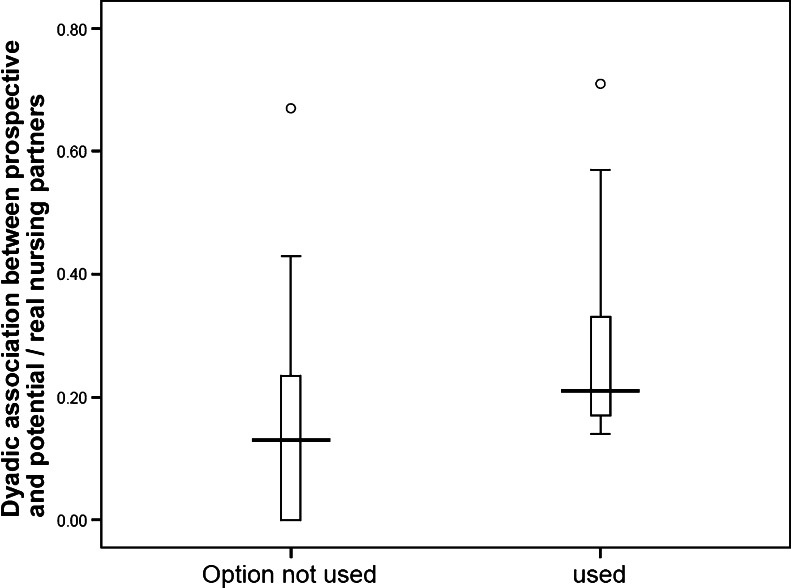
Individual associations of prospective mothers with the option to nurse communally towards unused and used (chosen) nursing partners, prior to the mothers' reproduction

Using females that overlapped for at least 5 days, we found that mothers had a significantly higher index of spatial association during the non-reproductive period with their actual nursing partners than with their potential, but not chosen, nursing partners (Fig. 1; *U* = 64.0, *N*_*1*_ = 27, *N*_*2*_ = 9, *P* = 0.036). Interestingly, the maximum association was similar in both groups.

### Determinants of communal nursing decisions

**Fig. 2:**
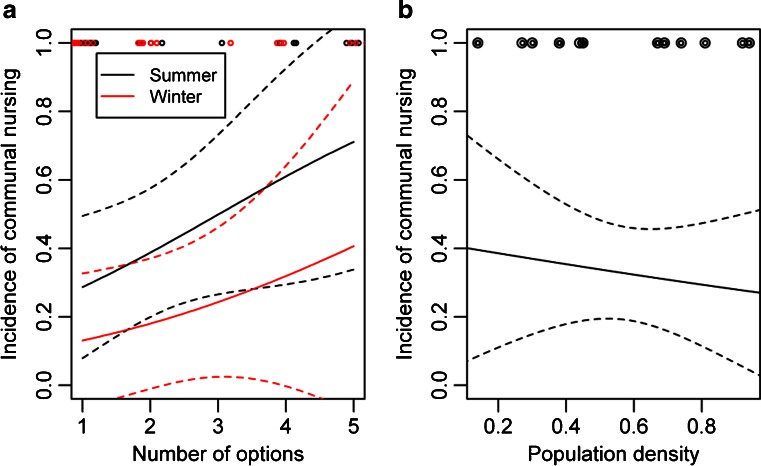
Incidence of communal nursing depending on a number of communal nursing options in summer and winter, and b population density

The incidence of communal nursing rose with the number of communal nursing options, and in summer compared to winter, but decreased with population density (Table 1, Fig. 2). The number of available communal nursing options was neither significantly correlated with density (Spearman's rho: 0.113, *P* = 0.391, *N* = 61), nor influenced by season (*U* = 263, *N*_*1*_ = 30, *N*_*2*_ = 19, *P* = 0.625).

**Table 1:** GLM analysis of incidence of communal nursing

Coefficient	Odds ratio estimate	95 % confidence interval	*z*_1,43_	*P* value
Number of options	0.651	0.532–0.965	2.332	**0.020**
Density	0.017	0.001–0.417	−1.968	**0.049**
Primiparous (yes vs. no)	0.819	0.309–0.931	0.977	0.329
Post-partum gestation (yes vs. no)	0.494	0.061–0.768	−0.716	0.474
Season (winter vs. summer)	0.214	0.016–0.445	−2.026	**0.043**

## Discussion

We predicted that if communal nursing is a by-product of group living, females would communally nurse whenever a litter had been born to another female with overlapping nest box use. We found that in only 33 % of cases when females had the opportunity to join a litter did they do so. Indeed, more than once, we observed two females, with overlapping home ranges, rearing litters solitarily at the same time in neighbouring nest boxes. Furthermore, the occurrence of communal nursing declined with population density. These results fail to support the hypothesis that communal nursing is non-adaptive.

Our evidence argues instead that females are selective. In a previous laboratory experiment (Weidt et al. 2008), female house mice that had strong spatial associations prior to reproduction were more likely to have egalitarian outcomes in communal nursing, and thus higher reproductive success. Consequences of a poor partner choice included reproductive failure through infanticide. In the present study, high-spatial association among communally nursing females prior to reproduction also predicted which partner was chosen. We further observed that females that nursed solitarily were not socially isolated, and that the probability of communal nursing increased with the number of available partners, suggesting that having more choices enhances the probability of finding a suitable partner. It is interesting to note that the occurrence of communal nursing was influenced by season and that communal nursing decreased with increasing population density. Further analyses should investigate whether these effects can be explained by seasonal differences in energy allocation during reproduction or by female competition. Female reproductive competition varies seasonally in this population and may cause the high reproductive skew observed (König and Lindholm 2012).

In conclusion, we reject the hypothesis that communal nursing is a non-adaptive by-product of group living. Our results indicate instead that females are choosy; suggesting that communal nursing with the right partner is adaptive.

## Electronic supplementary material

Below is the link to the electronic supplementary material.

ESM 1 (DOCX 18 kb)
