# Supplementary material for: Communal nursing in wild house mice is not a by-product of group living: Females choose
Source: Naturwissenschaften. 2014 Jan 4;101(1):73–6. doi: 10.1007/s00114-013-1130-6 (PMC3893474; doi:10.1007/s00114-013-1130-6)
Supplement: Supplementary file 1 — (DOCX 18 kb) [file 114_2013_1130_MOESM1_ESM.docx]

**SUPPLEMENTARY MATERIAL**

**Communal nursing in wild house mice is not a by-product of group living: females choose**

Andrea Weidt, Anna K. Lindholm & Barbara König

Institute of Evolutionary Biology und Environmental Studies, University of Zurich, Winterthurerstrasse 190, CH-8057 Zurich, Switzerland

Corresponding author: Barbara König, [barbara.koenig@ieu.uzh.ch](mailto:barbara.koenig@ieu.uzh.ch), +41 44 6355271

*Naturwissenschaften*

**Supplementary Methods**

*Reproduction and communal nursing*

Of a total of 106 litters, we assigned 92 litters (87%) to 38 mothers. Seventy-two of those assigned litters (68%) were reared in single nests, whereas 34 (32%) were reared in a communal nest. Of the 34 litters, 20 were in communal nests of two litters, 10 in nests of three litters, and 4 in nests of four litters (i.e. joined a nest of 3 litters). Female reproduction was skewed, with 157 adult females that did not produce litters.

*Spatial synchrony*

In the few litters where the mother was not tagged (N= 9) or where the litter could not be assigned to a mother (N=14), we could not determine spatial synchrony. In those cases we used the quarter where a litter was born (the barn was divided into quarters by aluminium dividers, which did not however prevent access to any part of the building) to determine communal nursing options because all reproducing females giving birth in the same quarter always used at least one common nest box.

*Fager’s index*

The Fager’s index gives the ratio between the number of days where both females were simultaneously found at the same nesting site and the number of days where both females were present in the barn, with a requirement of at least 5 days overlap. Analysis was only possible for litters and communal nursing options when mother assignments were resolved and when females were tagged and we therefore had available spatial information.
